# Novel *Corynebacterium diphtheriae* in Domestic Cats

**DOI:** 10.3201/eid1604.091107

**Published:** 2010-04

**Authors:** Aron J. Hall, Pamela K. Cassiday, Kathryn A. Bernard, Frances Bolt, Arnold G. Steigerwalt, Danae Bixler, Lucia C. Pawloski, Anne M. Whitney, Masaaki Iwaki, Adam Baldwin, Christopher G. Dowson, Takako Komiya, Motohide Takahashi, Hans P. Hinrikson, Maria L. Tondella

**Affiliations:** Centers for Disease Control and Prevention, Atlanta, Georgia, USA (A.J. Hall, P.K. Cassiday, A.G. Steigerwalt, L.C. Pawloski, A.M. Whitney, H.P. Henrikson, M.L. Tondella); West Virginia Department of Health and Human Resources, Charleston, West Virginia, USA (A.J. Hall, D. Bixler); Public Health Agency of Canada, Winnipeg, Manitoba, Canada (K.A. Bernard); University of Warwick, Coventry, UK (F. Bolt, A. Baldwin, C.G. Dowson); National Institute of Infectious Diseases, Tokyo, Japan (M. Iwaki, T. Komiya, M. Takahashi)

**Keywords:** Diphtheria, Corynebacterium, domestic cats, zoonoses, diphtheria toxin, *rpoB* sequencing, 16S rRNA sequencing, bacteria, dispatch

## Abstract

Novel nontoxigenic *Corynebacterium diphtheriae* was isolated from a domestic cat with severe otitis. Contact investigation and carrier study of human and animal contacts yielded 3 additional, identical isolates from cats, although no evidence of zoonotic transmission was identified. Molecular methods distinguished the feline isolates from known *C. diphtheriae*.

The clinical relevance of *Corynebacterium diphtheriae* recovered from a cat with otitis is poorly understood. Historically, humans have been thought to be its sole reservoir, and the few human cases reported annually in the United States are generally associated with international travel (*1*). Therefore, when *C. diphtheriae* was isolated from the ears of a cat, an investigation was initiated to evaluate potential sources of the cat’s infection and potential public health risks and to preliminarily characterize the *C. diphtheriae* isolate.

The cat, an 8-month-old female domestic shorthair, was examined at a West Virginia veterinary hospital on 5 occasions during January–June 2007. Pertinent findings included severe bilateral otitis, vestibular signs, mild ataxia, anorexia, and failure to gain weight; the cat had a history of ear, eye, and lung infections. Results of diagnostic tests showed no evidence of systemic disease and were negative for feline immunodeficiency and leukemia viruses and feline infectious peritonitis. Culture of an otic swab collected from the cat in May 2007 yielded 4 organisms: *C. diphtheriae*, *Streptococcus equi zooepidemicus*, *Staphylococcus* spp., and *Achromobacter xylosoxidans*. The cat was treated with oral clindamycin, otic enrofloxacin, and an ear-flushing solution.

## The Study

In June 2007, investigators visited the veterinary clinic and the household of the index cat and conducted a contact investigation and carrier study. Interviews of 2 household members and 8 veterinary staff members indicated no recent respiratory illness, skin infection, or risk factors for diphtheria (e.g., travel to countries to which diphtheria is endemic or contact with known case-patients). Half of these 10 contacts had received diphtheria vaccination within the previous 5 years. Cultures of oropharyngeal swab samples obtained from each person were negative, including cystine tellurite blood agar, which is selective for *C. diphtheriae*. Household members also were interviewed about medical history of a convenience sample of household animals (4 cats, including the index cat; 2 dogs; and 1 horse). Each animal was briefly examined, and oropharyngeal, otic, or ocular swab samples were collected. Otitis was observed in all 4 cats and 1 dog. The horse reportedly had had an eye infection ≈5 years earlier. No other abnormal findings were noted. Animal specimens yielded 3 additional isolates of *C. diphtheriae*: 1 from each ear of the index cat and 1 from the left ear of a 2-year-old domestic medium-hair cat. Both cats had been born on the premises and had remained with the same household since birth.

Feline *C.* *diphtheriae* and reference isolates used are described in the Table. Tinsdale agar plate growth (Remel, Lenexa, KS, USA) gave rise to black colonies with a brown halo, typical of cysteinase-producing *C.* *diphtheriae*, *C. ulcerans*, or *C. pseudotuberculosis*. After 24 hours on blood agar, 1–2-mm grey-white or opaque, rounded, convex colonies with no hemolysis were observed. Microscopically, the bacteria were gram-positive, club-shaped rods, 1 µm in diameter, arranged singly or at angles. Biochemical profiles to determine species and biotype were done by using an API Coryne strip (bioMérieux, Durham, NC, USA, and St-Laurent, Quebec, Canada). Query of API Coryne code 0010304 obtained for all isolates by APIWEB (https://apiweb.biomerieux.com) indicated a decreased level of confidence of *C. diphtheriae* biotype *mitis* or *belfanti* (89.5%) because of a maltose-negative result. Isolates were further characterized morphologically and biochemically by using tube substrates (*2*) and were identified by using a standard taxonomic scheme (*3*). Feline isolates were biochemically identical with each other and phenotypically consistent with *C. diphtheriae* biotype *belfanti*, except for the lack of maltose fermentation, which was considered an unusual finding (*3*).

**Table:** Feline *Corynebacterium diphtheriae* isolates and reference strains used for comparison, West Virginia, 2008*

Strain	Culture collection	Source	Diphtheria toxin	GenBank accession no.		
				16S rRNA	*rpoB*	*tox* gene
CD443	ATCC BAA-1774	Cat 1, right ear	Nontoxigenic	FJ409572	FJ415317	FJ376656
CD448	ND	Cat 1, right ear	Nontoxigenic	FJ409573	ND	FJ422272
CD449	ND	Cat 1, left ear	Nontoxigenic	FJ409574	ND	FJ422273
CD450	ND	Cat 2, left ear	Nontoxigenic	FJ409575	FJ415318	FJ422274
*C. diphtheriae* biotype *mitis*	NCTC 10356†	Human nose	Nontoxigenic	GQ118340	GQ409648	ND
*C. diphtheriae* biotype *gravis*	NCTC 10648	Unknown	Toxigenic	ND	ND	ND
*C. diphtheriae* biotype *gravis*	NCTC 11397^T^ ATCC 27010^T^	Unknown	Nontoxigenic	GQ118341	GQ409649	ND
*C. diphtheriae* biotype *gravis*	NCTC 13129 ATCC 700971	Human throat	Unknown	GQ118344	GQ409650	ND
*C. pseudotuberculosis*	NCTC 3450^T^	Sheep gland	Unknown	GQ118342	GQ409651	ND
*C. ulcerans*	NCTC 12077	Human throat	Unknown	GQ118343	ND	ND
*C. ulcerans*	NCTC 7910	Human throat	Unknown	GQ118345	ND	ND

*CD, Centers for Disease Control and Prevention identifier number; ATCC, American Type Culture Collection; ND, not deposited in this study; NCTC, National Collection of Type Cultures, London, UK. Additional strains used as controls for specific assays: toxigenic *C. diphtheriae* biotype *belfanti* isolates used for real-time PCR of *tox* gene were 718, G4182, C59, C60, C75, C76, C77; toxigenic *C. diphtheriae* ATCC 27012 used as positive control for Elek; *C. diphtheriae* NCTC 10481 and *C. ulcerans* CD199 used as positive and negative controls for Vero cell assay. †NCTC 10356 is described in the NCTC catalogue as *C. diphtheriae* biotype *mitis*; however, analyses in this study found this strain to be nitrate negative and therefore consistent with *C. diphtheriae* biotype *belfanti*. Thus, it was used in this study as a *belfanti* reference strain.

Antimicrobial drug susceptibility testing was performed according to the Clinical and Laboratory Standards Institute’s recommended methods and interpretative criteria (*4*). All 4 feline isolates were sensitive to ampicillin, cefepime, cefotaxime, ceftriaxone, cefuroxime, chloramphenicol, ciprofloxacin, clindamycin, daptomycin, erythromycin, ertapenam, gatifloxacin, gentamicin, levofloxacin, linezolid, meropenem, moxifloxacin, penicillin, quinupristin/dalfopristin, rifampin, telithromycin, tetracycline, tigecycline, trimethoprim/sulfamethoxazole, and vancomycin. Cellular fatty acid composition analysis was performed as described (*5*) by using the Sherlock system (MIDI, Inc., Newark, DE, USA), except that version 4.5 of the operating software was used. The cellular fatty acid composition profiles were consistent for *C. diphtheriae, C. ulcerans*, or *C. pseudotuberculosis*, including a substantial proportion (28%–30% of total) of C16:1ω7c (*5*). All feline isolates produced 7–15 meq/L of propionic acid among fermentation products, a feature associated with C*. diphtheriae* (*2*).

Results from use of the modified Elek test (*6*) indicated that all feline isolates were negative for production of diphtheria toxin; however, an atypical precipitation was observed after 36 h of incubation. Lack of toxin expression was corroborated by negative Vero cell assay results (*7*) and confirmed by using Western blot. Real-time PCR selective for the *C. diphtheriae* and *C. ulcerans* toxin gene (*tox*) (*8*) was positive for all feline isolates. However, real-time PCR for A and B subunits of *tox* (*9*) amplified subunit A but not subunit B. Sequence analysis of the *tox* gene was performed as previously outlined (*10*) and compared with a reference *tox* gene, GenBank accession no. K01722. The 4 feline *tox* sequences were identical to each other but contained multiple nucleotide substitutions and deletions compared with the reference gene. By NCBI BLAST search (http://blast.ncbi.nlm.nih.gov/Blast.cgi), the feline *tox* had higher sequence identity (97%–98%) to the *tox* sequences of *C. ulcerans*, compared with those from C. diphtheriae (94%–95%). A deletion at nt 55, coupled with a cytosine-to-thymine substitution at nt 74, prematurely terminated the peptide at aa 25.

**Figure 1:**
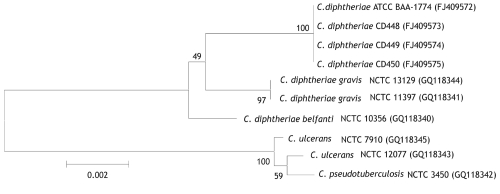
Neighbor-joining phylogenetic tree based on 16S rRNA gene sequence analysis of *Corynebacterium diphtheriae* isolates, including 4 feline isolates from West Virginia, 2008 (ATCC BAA-1774, CD 448, CD 449, CD 450). The tree was constructed from a 1,437-bp alignment of 16S rRNA gene sequences by using the neighbor-joining method and Kimura 2-parameter substitution model. Bootstrap values (expressed as percentages of 1,000 replicates) >40% are illustrated at branch points. Feline isolates had 100% identity with each other and >99.1% identity with *C. diphtheriae* biotypes *gravis* and *belfanti*. GenBank accession nos. given in parentheses. ATCC, American Type Culture Collection; CD, Centers for Disease Control and Prevention identifier number; NCTC, National Collection of Type Cultures. Scale bar indicates number of substitutions per site.

**Figure 2:**
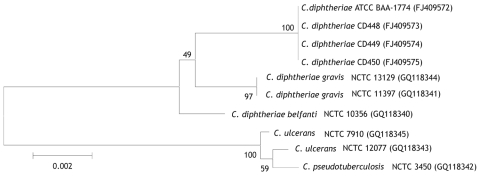
Jukes-Cantor–derived phylogenetic tree based on sequence analysis of a selected region of the *rpoB* gene of *Corynebacterium* isolates, including 2 feline isolates from West Virginia, 2008 (ATCC BAA-1774, CD 450). Feline isolates had 100% identity with each other and 97.7% identity with *C. diphtheriae* biotypes *gravis* and *belfanti*. GenBank accession nos. given in parentheses. ATCC, American Type Culture Collection; CD, Centers for Disease Control and Prevention identifier number; NCTC, National Collection of Type Cultures. Scale bar indicates number of substitutions per site.

Species characterization was corroborated by using 16S rRNA (*11*) and partial *rpoB* (*12*) gene sequencing. By 16S rRNA gene sequence analysis, the feline strains had 100% identity with each other and >99.1% identity with various reference sequences for *C. diphtheriae* biotype *gravis* and *belfanti* sequences, including NCTC 11397^T^. Partial *rpoB* sequence analyses indicated 100% identity among the feline isolates and 97.7% identity with *C. diphtheriae* NCTC 11397^T^. Neighbor-joining phylogenetic trees based on both 16S rRNA (Figure 1) and partial *rpoB* gene sequencing (Figure 2) positioned the feline isolate sequences within the *C. diphtheriae* clade but clearly distinguished them from the other *C. diphtheriae* isolates. Comprehensive molecular analyses to characterize differences between biotype *belfanti* strains, including these feline isolates, with other *C. diphtheriae* biotypes, are the subject of a separate publication (C.G. Dowson, pers. comm.).

## Conclusions

We identified a potentially novel biotype of *C. diphtheriae* recovered from domestic cats in West Virginia but found no evidence of zoonotic transmission. Although rare, isolation of *C. diphtheriae* from animals has been reported, including *C. diphtheriae* biotype *belfanti* from a skin lesion of a cow (*13*) and toxigenic *C. diphtheriae* biotype *gravis* from a wound of a horse (*14*). *C. ulcerans* is a known animal pathogen, and zoonotic transmission of toxigenic *C. ulcerans* from companion animals has been reported, often associated with predisposing concurrent illnesses (*15*).

The feline strains isolated during this investigation differed phenotypically from previously described biotypes but were otherwise regarded as typical of *C. diphtheriae*. However, isolates were nontoxigenic and harbored a modified *tox* gene with sequence differences from *Corynebacterium* spp. capable of expressing diphtheria toxin. On the basis of published criteria (*11*), the feline strain might represent a novel subspecies of *C. diphtheriae* because it shares <98% sequence homology to the type strain within the *rpoB* gene. Potential for zoonotic transmission of this novel, cat-associated *C. diphtheriae* and associated public health implications are unknown. Additional studies are needed to further characterize these isolates and determine their appropriate taxonomy. Large-scale screening of domestic cat populations is recommended to determine the prevalence of *C. diphtheriae* and its pathogenic potential and to identify additional isolates for more formal description and classification.
